# Dicodon-based measures for modeling gene expression

**DOI:** 10.1093/bioinformatics/btad380

**Published:** 2023-06-12

**Authors:** Andres M Alonso, Luis Diambra

**Affiliations:** Instituto Tecnológico Chascomús (INTECH), CONICET-UNSAM, Intendente Marino km 8.2, Chascomús, 7130 Provincia de Buenos Aires, Argentina; CCT-La Plata, CONICET, Calle 8 Nº 1467, La Plata, B1904CMC Provincia de Buenos Aires, Argentina; CCT-La Plata, CONICET, Calle 8 Nº 1467, La Plata, B1904CMC Provincia de Buenos Aires, Argentina; Centro Regional de Estudios Genómicos, FCE-UNLP, Blvd 120 N∘ 1461, La Plata, 1900 Provincia de Buenos Aires, Argentina

## Abstract

**Motivation:**

Codon usage preference patterns have been associated with modulation of translation efficiency, protein folding, and mRNA decay. However, new studies support that codon pair usage has also a remarkable effect at the gene expression level. Here, we expand the concept of CAI to answer if codon pair usage patterns can be understood in terms of codon usage bias, or if they offer new information regarding coding translation efficiency.

**Results:**

Through the implementation of a weighting strategy to consider the dicodon contributions, we observe that the dicodon-based measure has greater correlations with gene expression level than CAI. Interestingly, we have noted that dicodons associated with a low value of adaptiveness are related to dicodons which mediate strong translational inhibition in yeast. We have also noticed that some codon-pairs have a smaller dicodon contribution than estimated by the product of the respective codon contributions.

**Availability and implementation:**

Scripts, implemented in Python, are freely available for download at https://zenodo.org/record/7738276#.ZBIDBtLMIdU.

## 1 Introduction

The study of gene expression can explain how protein production is regulated by different molecular entities that interpret genome information. However, in addition to those regulatory molecules, protein level can partly be explained by the codon usage pattern in the coding sequences (Fredrick and Ibba 2010). These patterns arise from the redundancy of the genetic code since this property provides the possibility to use synonymous codons differentially (Kurland 1991). The preference in codon usage, known as codon usage bias (CUB), is specific to each organism, but species that are close to each other often show a similar codon usage preference pattern (Plotkin and Kudla 2011). CUB has been associated with modulation of translation efficiency, protein folding, and mRNA decay in a species-specific manner (Hanson and Coller 2018). Therefore, CUB has numerous applications such as gene prediction (Burge and Karlin 1997), protein–protein interaction (Fraser *et al.* 2004), and heterologous gene expression (Gustafsson *et al.* 2004).

Available data from gene expression analysis experiments, like microarrays or RNA-seq, are great resources to explore translational efficiency and codon usage patterns in different organisms (Lithwick and Margalit 2005, Hershberg and Petrov 2008, Plotkin and Kudla 2011). In this manner, statistical analysis of this available information makes it possible to study different kinds of measures to quantify codon bias in order to characterize the optimality of each codon. Among these statistical measures, we can mention the relative synonymous codon usage (Sharp and Li 1986), the relative adaptiveness (Sharp and Li 1987), frequency of optimal codons (Ikemura 1981), the codon bias index (Bennetzen and Hall 1982), and the expression measure (Karlin and Mrázek 2000). Most of them are based on the codon usage frequencies on a set of highly expressed genes and they were recently reviewed in (Bahiri-Elitzur and Tuller 2021). The selection of a set of highly expressed genes is supported by the fact that those genes are under strong translational selection and the synonymous codons that compose those have hard selective constraints, However, this is a strong assumption, because the factors shaping the codon preference in a genome are, in general, species dependent.

Researchers have also devised some functions that map codon usage measures to a single number or index, which can be used for optimizing the heterologous expression of genes in foreign hosts (Sharp and Li 1987, Roymondal *et al.* 2009, Fox and Erill 2010). Most of these indexes use the geometric mean, introduced by Gribskov *et al.* (1984). One of the most used examples of these measures is CAI (Sharp and Li 1987), which is defined as the geometric mean of the codon’s relative adaptiveness. Of course, the relative adaptiveness depends on the codons but not on their positions. In general, these codon-based indexes present some correlation with expression levels in many organisms.

The main advantage of these indexes is that they are very simple to calculate because they only involve computing the frequency of codons in a reference gen set. However, in addition to not being sensitive beyond a single codon distribution, they have other disadvantages (Salim and Cavalcanti 2008). For example, they may miss specific regulator factors of gene expression such as secondary structure and nucleotide composition. Further, it is mandatory to choose the reference genes which may include many biases; i.e. some measures can be influenced by the length of the coding sequence (Ingvarsson 2007). Usually, the results obtained with different reference gene sets vary substantially.

On the other hand, early studies consider codon pair usage as a phenomenon that regulates protein translational process at the level of fidelity and efficiency (Gutman and Hatfield 1989, Irwin *et al.* 1995). In fact, a bias on using two successive codons, hereafter dicodons, is a well-studied issue in various organisms and is supported by experimental evidence (Fedorov 2002, Tats *et al.* 2008, Guo *et al.* 2012, Gamble *et al.* 2016, Kunec and Osterrieder 2016, Diambra 2017). For example, a comparative analysis of the codon pair usage confirms that certain dicodons are avoided since others are preferred in the ORFeome of the analyzed genomes (Tats *et al.* 2008). In addition, by using a yeast approach to estimate the expression of 35 811 GFP variants, with three random adjacent codons, the authors identified 17 dicodons associated with strong inhibition of translation (Gamble *et al.* 2016). Furthermore, a marked difference in codon pair frequency is observed when coding sequences from high-abundance proteins are compared to coding sequences from low-abundance proteins in model organisms (Diambra 2017). Also, hidden Markov models for gene prediction based on hexamers frequency have better accuracy than models based on codons (Burge and Karlin 1998, Korf *et al.* 2001). Based on these findings, it can be hypothesized that the translation performance of the sequence could be better described in terms of dicodons. However, strategies for mapping coding sequences to expression indexes based on dicodons usage frequencies have not been explored yet.

In this work, we extend the geometric mean based-index by considering the contributions of dicodons on the nucleotide sequences. Alternatively, we also introduce a new way to measure the contributions, taking into account the expression level for each sequence. Thus, using the traditional no-weighted and the new weighted contributions we compute through the geometric mean both, codon- and dicodon-based indexes to evaluate their correlations with gene expression level. Our results show that dicodons are more informative than codons and could be used to design new biotechnological applications, like the design of attenuated virus (Coleman *et al.* 2008) and the improvement of protein heterologous expression to a rational design of transcripts that reduce protein misfolding (Mauro 2018, Papamichail *et al.* 2018).

## 2 Materials and methods

### 2.1 Data description

In this work, we have used two kinds of data across four organisms: (i) transcript abundance and (ii) nucleotide sequences associated with the coding regions. The transcriptome profiles corresponding to *Escherichia coli* and *Saccharomyces cerevisiae*, were downloaded from the Many Microbe Microarrays database (http://m3d.mssm.edu) (Faith *et al.* 2008). From *E.coli*, we selected 26 transcriptome profiles associated with wild-type studies obtained by microarray. From *S.cerevisiae* we select 61 transcriptome profiles, obtained by microarray, associated with three GEO series: GSE3076 [16 conditions with 3 biological replicates (BR)] from Guan *et al.* (2006), GSE3431 (36 conditions with 1 BR) from Tu *et al.* (2005), and GSE4807 (9 conditions with 3 BR) from Knijnenburg *et al.* (2007). We also included transcriptome profiles corresponding to the Apicomplexa *Toxoplasma gondii* and to the fruit fly Drosophila *melanogaster*. In the case of *T.gondii*, we consider transcriptome profiles associated with 10 conditions: tachyzoite 24 h postinfection (Waldman *et al.* 2020), tachyzoite infection in four mouse cell types (Swierzy *et al.* 2017), rat nontransformed epithelial cell line IEC-18 infection (Guiton *et al.* 2017), tachyzoite 3–4 days postinfection (Reid *et al.* 2012) and two conditions corresponding to acute and chronic infection in mouse (Pittman *et al.* 2014). All these datasets were obtained by RNAseq and we downloaded the normalized values [transcripts per million (TPM)] from *T.gondii* database (Gajria *et al.* 2007). In the case of *D.melanogaster*, we consider the transcriptome profiles, obtained by microarray, from five study series: GSE3955 (five conditions with three BR) from Pilot *et al.* (2006), GSE6515 (one condition with five BR) from Magalhães *et al.* (2007), GSE7763 (11 conditions with 3 BR) from Baker *et al.* (2007), GSE9149 (two conditions with three BR) from Chintapalli *et al.* (2012) and E-MEXP-2580 (three conditions with four BR) from Thomsen *et al.* (2010). These studies correspond to the wild-type fly and were downloaded from Bgee database (http://bgee.org) (Bastian *et al.* 2021).

All databases above provide mean transcriptome profiles, i.e. the biological replicates were averaged. In addition in the case of microarray studies, the mean expression profiles downloaded from databases correspond to log-normalized expression values, for that reason we need to take the exponential of these values before they are considered as the expression level. Detailed information about each study, condition, biological replicates, and links is listed in Supplementary Table S1.

From the Ensembl website for eukaryote organisms, we downloaded the nucleotide coding sequences corresponding to yeast (ftp.ensemblgenomes.ebi.ac.uk/pub/fungi/release-56/fasta/saccharomyces_cerevisiae/cds/Saccharomyces_cerevisiae.R64-1-1.cds.all.fa.gz) and fruit fly (ftp.flybase.net/genomes/Drosophila_melanogaster/dmel_r6.30_FB2019_05/fasta/dmel-all-CDS-r6.30.fasta.gz). The nucleotide coding sequences corresponding to *E.coli* were downloaded from Ensembl website for prokaryote organisms (ftp.ensemblgenomes.ebi.ac.uk/pub/bacteria/release-56/fasta/bacteria_79_collection/escherichia_coli_str_k_12_substr_w3110_gca_000010245/cds/Escherichia_coli_str_k_12_substr_w3110_gca_000010245.ASM1024v1.cds.all.fa.gz), while coding sequences corresponding to *T.gondii* genes were downloaded from ToxoDB database (Gajria *et al.* 2007). We have restricted our analysis to coding sequences with lengths >50 codons, disregarding the first codon and first dicodon. Stop codons and dicodons that contain a stop codon are not considered for further analysis. The coding sequences and transcript profiles used in our study are also available for download at https://zenodo.org/record/7738276#.ZBIDBtLMIdU.

### 2.2 The measures

An expression index is a function that maps a nucleotide sequence into the associated gene expression level. Most of these codon preference statistics use the geometric mean of the contributions associated with the codons that make up the sequence (Gribskov *et al.* 1984), defined as where $C_{c}(i)$ is the contribution of the codon *c* located at position *i* and *L* is the length of the sequence considered. In this paper, we use four different codon contributions that can be classified according to whether they are based on codons or dicodons. They can also be classified according to whether, or not, they use a weighting strategy according to the level of expression of the sequence. The one based on codons without a weighting strategy corresponds to the well-known CAI (Sharp and Li 1987).


(1) $$\text{Expression}\;\text{index}={\left(\prod_{i}^{L}C_{c}(i)\right)}^{1/L},$$

### 2.3 Contributions without weighting strategy

Using a set of sequences *S* as input, we count the observed number of codons and dicodons, *o*_c_ and *o*_d_, respectively. Then, we use these counts to calculate the relative codon adaptiveness (Sharp and Li 1987), *a*_c_, and its extension for dicodons, *a*_d_, which are defined as: where $\max\{o_{c}\}$ and $\max\{o_{d}\}$ are the counts of the most frequent synonymous codon of c and dicodon d, respectively. Codons or dicodons with relative adaptiveness equal to one can be considered translationally optimal.


$$a_{c}=\frac{o_{c}}{\max\{o_{c}\}},\;\;\;\;\;\;a_{d}=\frac{o_{d}}{\max\{o_{d}\}},$$

### 2.4 Contributions with weighting strategy

In this case, we define a dicodon contribution *b*_d_ where the observed count of dicodons in a set of *N* sequences $S=\{s_{1},s_{2},\ldots ,s_{N}\}$ is weighted with a magnitude relative to the expression level of the sequence *s_i_*, i.e. where $o_{d}(s_{i})$ is the count of dicodon *d* in each *s_i_* belonging to *S*. The weight $w(s_{i})$ can be defined in terms of the transcript or protein abundance levels associated with sequence *s_i_*, depending on the data availability. Of course, this summation (2) must be normalized. Thus, the relative weighted contribution of dicodon *d* can be expressed as the ratio $f_{d}=b_{d}/(\max\{b_{d}\})$, where $\max\{b_{d}\}$ is the maximum of the weighted counts *b*_d_ associated to all synonymous dicodons of *d*. The weighted contribution defined above can also be defined for codons. In fact, we also performed this computation for the sake of comparison and this codon-weighted contribution will be denoted by *f*_c_.


(2) $$b_{d}=\sum_{i=1}^{N}o_{d}(s_{i})w(s_{i}),$$

### 2.5 The sets of sequences and weights

The effect of different reference sets used to compute the codon contribution has been studied in many papers (Supek and Vlahoviček 2005, Fox and Erill 2010, Roth *et al.* 2012, Hanson and Coller 2018). In general, high-quality transcript/protein abundance data are required to define a suitable reference set. In this paper, we count codons and dicodons over sequence sets *S_p_* that include genes with expression levels above the percentile *p*, using different percentile values p=99,97,95,90,80 and also 0, which indicates that all sequences available are used.

In the case of *S.cerevisiae*, *E.coli*, and *D.melanogaster*, where expression levels are log-normalized, the weights are defined as $w(s_{i})=\text{exp}(E_{i})$ and *E_i_* is the log-normalized expression level of gene *i*. On the other hand, in the case of *T.gondii*, weights are defined as $w(s_{i})=E_{i}$, where *E_i_* is expression level of gene *i* in TPM.

## 3 Results

**Figure 1.**
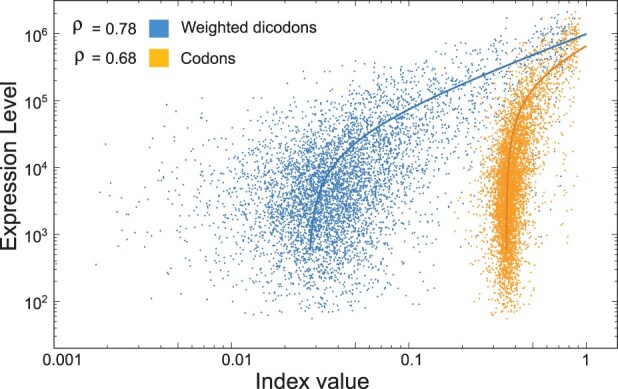
Raster plot of two expression indexes versus expression levels in yeast, considering the weighted dicodon-based scheme (left curve) and codon-based scheme (right curve). In both cases, the expression indexes were obtained using sequences with expression levels greater than the 97 percentile. Solid lines correspond to linear regression lines.

**Figure 2.**
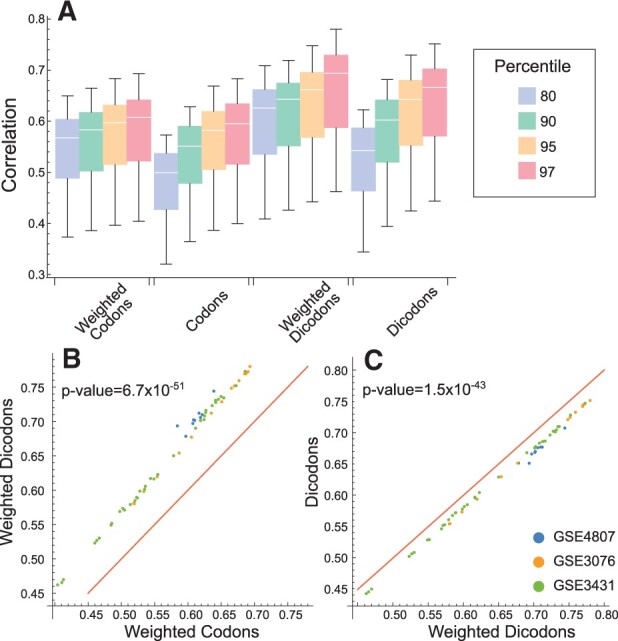
Pearson correlation coefficients between the expression indexes and expression level in yeast. They correspond to four schemes (weighted codons, no-weighted codons, weighted dicodons, and no-weighted dicodons) using transcript sequences with different percentiles (A). The correlation coefficients are computed for each sample independently. For comparison among schemes, we plot the correlations obtained for weighted codons versus weighted dicodons (B), and weighted dicodons versus no-weighted dicodons (C). Straight-line is the identity line and the *P*-values were obtained with the paired *T*-test. Similar comparisons for all studied organisms are depicted in Supplementary Figs S2–S5.

Using the traditional no-weighted and the new weighted contributions, we compute through the geometric mean both, codon- and dicodon-based indexes (see Table 1) and their correlation with the expression level. In Fig. 1, we depict a raster plot of the expression levels of *S.cerevisiae* transcriptome versus the dicodon expression index using weighted contributions and versus the codon expression index using weighted contributions. The *f*_d_ were obtained by counting over a sequence set *S*_97_ of one *S.cerevisiae* sample. Pearson’s correlation coefficient correlation associated with the plot is near 0.78. For the sake of comparison, in Fig. 2A we plot the correlations obtained for the expression index computed with the four contributions listed in Table 1 (61 yeast samples). All expression indexes were computed for four different reference gen sets: *S*_97_, *S*_95_, *S*_90_, and *S*_80_, and for each sample independently. Firstly, we observed that, independently of the reference gene set used, dicodon-based indexes have a greater correlation than codon-based indexes. This is evident in Fig. 2B which shows the correlations obtained with codon- and dicodon-based indexes for each one of the 61 experimental conditions of yeast with *S*_97_. Paired *T*-test indicates that means of correlations coefficient obtained from codon- and dicodon-based indexes, with the weighted strategy, are significatively different (*P*-value = 6.7 $\times 10^{-51}$).

**Table 1.** The new weighted and the traditional no-weighted contributions, both codon- and dicodon-based indexes.^a^

	Codon-based	Dicodon-based
No-weighted	$a_{c}=\frac{o_{c}}{\max\{o_{c}\}}$	$a_{d}=\frac{o_{d}}{\max\{o_{d}\}}$
Weighted	$f_{c}=\frac{b_{c}}{\max\{b_{c}\}}$	$f_{d}=\frac{b_{d}}{\max\{b_{d}\}}$

a Of course, the codon-based index without weight corresponds to the traditional CAI.

Furthermore, Fig. 2B compares the correlations obtained for weighted and no-weighted strategies for the dicodon-based expression indexes with *S*_97_. In this case, the paired *T*-test indicates that correlation coefficients obtained with the weighted strategy are significatively greater than the ones obtained with the no-weighted strategy (*P*-value = 1.5 $\times 10^{-43}$). Similar results were obtained by considering the Spearman-rank correlation as comparison metrics, but with higher *P*-values (Supplementary Fig. S1). These statistical analyses were performed for the four organisms considered here and the results are consistent with the premises: dicodon-based indexes have a better performance than codon-based indexes, and the weighted strategy improves performance with respect to the no-weighted strategy, in particular in the dicodon-based cases. We have performed the comparisons of the correlations coefficient obtained with different expression indexes schemes and reference gene sets (*S*_80_, *S*_90_, and *S*_97_) for *S.cerevisiae* (Supplementary Fig. S2), *E.coli* (Supplementary Fig. S3), *T.gondii* (Supplementary Fig. S4), and *D.melanogaster* (Supplementary Fig. S5). These results suggest that dicodons are more informative than codons to model expression levels from nucleotide sequences.

The CAI was originally defined with a set of genes empirically proven to be highly expressed in yeast and *E.coli* (Sharp and Li 1987). Other authors have added transcription/translation-related factors and chaperones in the reference set (Karlin and Mrázek 2000), and/or ribosomal protein genes (Supek and Vlahoviček 2005). In Fig. 2A one can observe, as a general behavior, that the correlations of indexes with expression levels decrease as the reference set includes more gene sequences with lower expression levels. The same behaviors are observed in the other three organisms, as shown in Supplementary Fig. S6. However, this is not true when considering the Spearman-rank metric instead of Pearson correlation, where in the case of the weighted dicodon index the performance obtained for *S*_95_ is better than *S*_97_ (*P*-value $=3.8\times 10^{-8}$, Supplementary Fig. S1).

**Figure 3.**
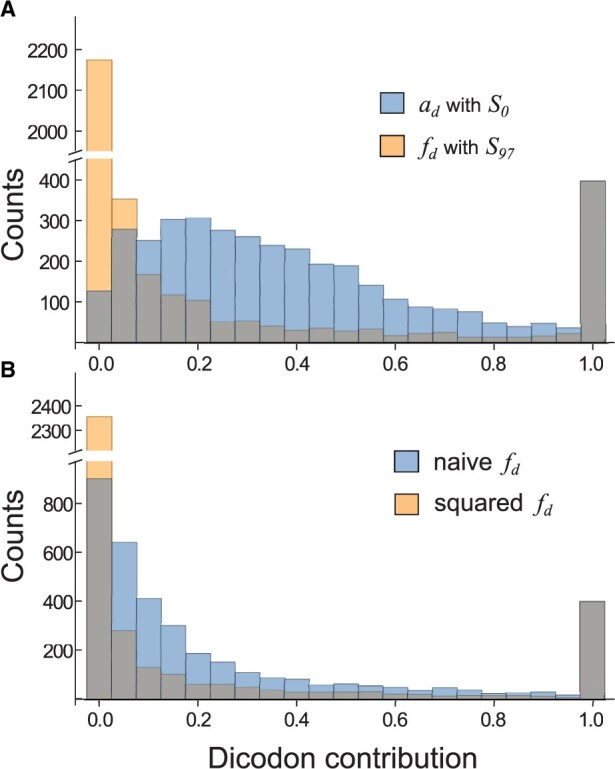
Histogram of the dicodon contributions, *f*_d_, obtained for the weighted case using sequences *S*_97_ (yellow bars) and for the no-weighted case, *a*_d_, using all transcript sequences *S*_0_ (blue bars) (A). Histogram of the dicodon contributions obtained for the weighted case using all sequences (blue bars) and for the case in which contributions were squared (yellow bars) (B). Values that superimpose are highlighted in gray.

We see that weighting the contributions according to the sequence abundance has a similar effect that selecting the sequence associated with highly expressed genes. In fact, one can understand this selection as a tight weighting procedure where all the selected sequences have the same weight, while the discarded sequences are weighted with a null weight. In order to understand the role of the contributions weighting we compute two histograms: (i) from dicodon contribution, *f*_d_, obtained by counting over a sequence set *S*_97_ using the weighting, and (ii) from dicodon contribution, *a*_d_, obtained by counting overall sequences (*S*_0_) using the not weighting schemes (yellow and blue bars, respectively, in Fig. 3A). This plot shows that there is a significant difference among the contributions associated with these schemes (Kolomogorov–Smirnov test, *P*-value ≤10^−300^), while not-weighted contributions are broadly distributed with a mode around 0.2 (blue bars), most of the weighted contributions are accumulated at lower values (yellow bars) and are in agreement with the number of dicodons that contribute with one. One can hypothesize that broad distributions could be associated with worse performance and a transformation of the contributions, according to this observation, can lead to an improvement in the performance. Figure 3B displays the histogram of dicodon contributions obtained by counting over all sequences (i.e. *S*_0_) using the weighting schemes (blue bars). Similarly to the weighting case of Fig. 3A, most of the dicodons are associated with small contributions. The distribution obtained when all contributions are squared (i.e. it is the histogram of $f_{d}^{2}$) is more biased to the extremes (yellow bars).

**Figure 4.**
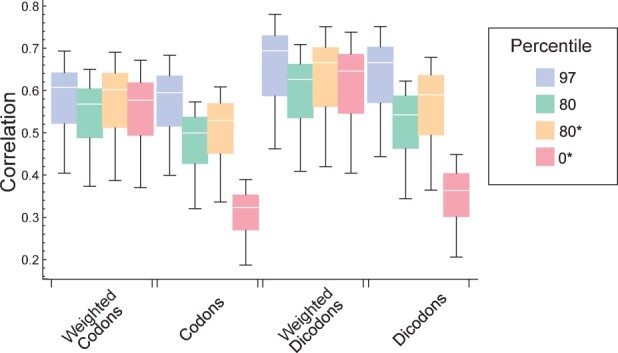
Pearson correlation coefficients between different expression indexes and the expression levels in yeast. They correspond to four schemes (weighted codons, no-weighted codons, weighted dicodons, and no-weighted dicodons) using different weights. The ones obtained with the sequence set *S*_97_ and *S*_80_ are the same that were shown in Fig. 2A. Last two boxes were obtained using squared weights from sequences set *S*_80_ and *S*_0_, respectively.

In Fig. 4, we depict the correlations obtained with the squared contributions in yeast, i.e.


(3) $$\text{expression}\;\text{index}={\left(\prod_{i}^{L}C^{2}(i)\right)}^{1/L}.$$

For the sake of comparison, we also included two cases also depicted in Fig. 2. When comparing the performances obtained for *S*_80_, clearly the square transformation increases the correlation (*P*-value = $2.4\times 10^{-35}$ for weighted dicodons). In the case of weighted schemes, this improvement allows a good performance even for contributions computed without selecting a set of high-expression sequences *S*_0_. However, even with this improvement, dicodon-based indexes are more efficient than codon-based indexes, and indices with weighted contributions are more efficient than those with the no-weighted strategy.

**Figure 5.**
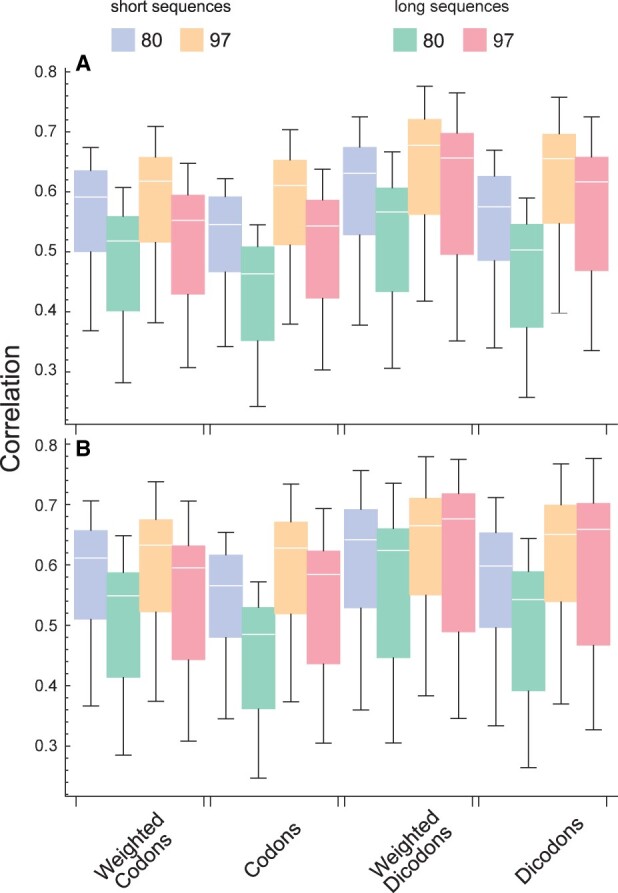
Pearson correlation coefficients for different expression indexes obtained from two groups of transcripts: short sequence and long sequence (A). The correlations were obtained for the same conditions as (A) but using squared weights (B).

So far as we have computed the correlation over all available sequences. However, in general, the correlation of any expression index depends on coding, but also on other factors like sequence length (Fox and Erill 2010, Bahiri-Elitzur and Tuller 2021). In order to study the stability of performances with the sequence length we examined the correlations with expression level by considering two sets of sequences that differ in their mean lengths: coding sequences with lengths above the percentile 80 (long sequences), and coding sequences below the percentile 20 (short sequences). Figure 5 depicts the correlations obtained with the four schemes listed in Table 1 computed over the short-sequences set (blue and yellow boxes) and over the long-sequences set (green and red boxes) for the yeast samples. In Fig. 5A, one can see that the correlations obtained for long sequences are smaller than the corresponding to short sequences in all cases. These differences are statistically significant, at the level of 0.01, in all cases with the exception of the dicodon-based measures using *S*_97_ (*P*-values of paired *T*-test are listed in Supplementary Table S2). This result suggests that dicodon-based measures decrease the biases between long and short sequences, particularly the weighted strategy. Further, Fig. 5B depicts the results from a similar analysis but using squared contributions for codons and dicodons. Again the correlations obtained for long sequences are smaller than the corresponding to short sequences. However, these differences are smaller than in Fig. 5A. In particular, they are not statistically significant for the dicodon-based measures using *S*_90_ and *S*_97_ (*P*-values listed in Supplementary Table S2). Thus, the comparison of results depicted in Fig. 5 suggests that squared transformation of contribution decreases the bias between long and short sequences.

**Figure 6.**
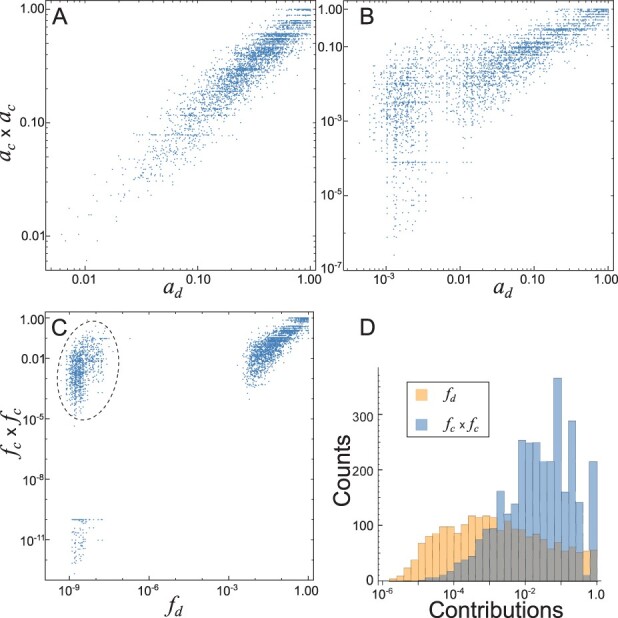
Raster plots of expected dicodon adaptiveness $a_{c}\times a_{c}$ versus dicodon adaptiveness *a*_d_ in yeast, obtained by using sequences set *S*_0_ (A) and *S*_97_ (B). Raster plot of expected dicodon contribution $f_{c}\times f_{c}$ versus dicodon adaptiveness *f*_d_ in yeast, obtained by using sequences set *S*_97_ (C). The dots in the dashed ellipse correspond to a set of dicodons whose contribution is clearly overestimated by the product $f_{c}\times f_{c}$. Histogram of the dicodon contribution, depicted in (C), obtained for *f*_d_ and for $f_{c}\times f_{c}$ (D).

**Figure 7.**
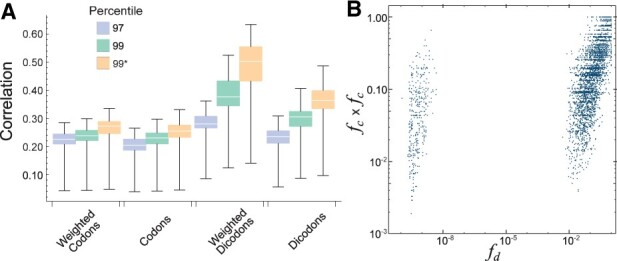
The correlation coefficients between the expression indexes and the expression level in *D.melanogaster* (A). They correspond to four schemes (weighted codons, no-weighted codons, weighted dicodons, no-weighted dicodons) using weights obtained with sequence sets *S*_97_ and *S*_99_. Yellow boxes were obtained using squared weights from sequences set *S*_99_. Raster plots of expected dicodon contribution $f_{c}\times f_{c}$ versus dicodon adaptiveness *f*_d_ in *D.melanogaster*, obtained by using sequences set *S*_99_ (B).

One reason for the out-performance of dicodon-based indexes seems to be that many dicodons’ contributions are close to zero and cannot be explained solely by the codon frequencies, especially when we restrict the analysis to the set of highly expressed sequences. In Fig. 6A and B, we depict raster plots of dicodon adaptiveness *a*_d_ in yeast versus an expected dicodon adaptiveness which is obtained by the product of each codon adaptiveness ($a_{c}\times a_{c}$), computed by using sequences set *S*_0_ and *S*_97_, respectively. In the latter, restricted to highly expressed sequences, we see that some dicodons depart from an expected linear relationship. This effect is more remarkable when we are dealing with weighted contributions *f*_d_, where there are clearly three clusters (Fig. 6C). The smaller cluster consists of only 121 dicodons and includes 11 (AGGCGA, ATACGA, CGAATA, CGACCG, CGACGA, CGACGG, CGACTG, CGAGCG, CTGCGA, GTACGA, GTGCGA) of the 17 dicodons which mediate strong translational inhibition in yeast according to Gamble *et al.* (2016). The second cluster, indicated by a dashed ellipse, is formed by 1026 dicodons and includes 4 (AGGCGG, ATACGG, CTCCCG, CTGCCG) of these 17 dicodons. The larger cluster contains the remaining dicodons and includes only two of the inhibitory dicodons (CTGATA, GTACCG). In fact, all these inhibitory dicodons, with the exception of the last two, have associated a very small *f*_d_ value ($<3.5\times 10^{-9}$). The dicodons with small *f*_d_ value, and not included in the 17 inhibitory dicodons are GCGCTC, GCGCTG, GCGGCA, GCGGGT, GCTCTC, GGCGGG, and GGGGCG, and could be considered for further translational inhibition studies. It is interesting to note that Fig. 6C depicts several dicodons whose contribution is clearly overestimated by the product $f_{c}\times f_{c}$ (dots inside the dashed ellipse). Many of dicodons’ contributions are accumulated at a lower value as we see in the histogram of contributions *f*_d_ (yellow bars) in contrast to $f_{c}\times f_{c}$ (blue bars). Although the existing expression indexes in the literature today present correlations with expressivity when applied to unicellular organisms, their performance is more elusive when applied to multicellular organisms. However, as we show in Fig. 7A, the correlations between expression levels and expression indexes based on dicodons are much greater than the results obtained with expression indexes based on codons. This improvement is almost 100% than in the case of *D.melanogaster* when the comparison is made on squared weights based on dicodons and *S*_99_ where the average correlation reaches 0.45. Further, in a similar manner than in yeast, Fig. 7B shows that there are dicodon contributions that are overestimated by the product $f_{c}\times f_{c}$. These results suggest that dicodon-based measures could be used to computationally identify dicodons associated with a strong inhibition of translation as in Gamble *et al.* (2016).

## 4 Discussion

Understanding CUB across species is important to improve our knowledge about gene expression (Gustafsson *et al.* 2004, Supek and Vlahoviček 2005), or phylogenomic inference (Christianson 2005, Shackelton *et al.* 2006). Because of the relevance of codon usage information across species, there exist several databases which provide these metrics (Nakamura *et al.* 2000, Alexaki *et al.* 2019, Subramanian *et al.* 2022). Additionally, a variety of studies based on different strategies have proposed measures with the aim of predicting protein expression from a coding sequence (Supek and Vlahoviček 2005, Burgess-Brown *et al.* 2008, Plotkin and Kudla 2011). More recently, researchers have reported that the usage frequency of adjacent codons pairs, or dicodons, have correlations that are not explained by the frequency of single codons (Diambra 2017). Furthermore, dicodons have been linked to ribosomal pauses and overall expression levels (Gamble *et al.* 2016). Consequently, it arises as an attractive field for gene prediction and to improve recombinant gene expression (Coleman *et al.* 2008, Chung *et al.* 2013, Huang *et al.* 2021).

In this work, we expand the codon adaptation index that is based on the frequency of single codons from a representative set of highly expressed genes (Sharp and Li 1987). We incorporate dicodon frequencies on a weighted strategy based on available expression data. When our strategy was applied for *E.coli*, yeast, *T.gondii*, and fruit fly, the results confirm that the new approach outperforms expression indexes based on single-codon strategies in all cases. In fact, the correlation coefficient between expression indexes and expression levels was better for yeast genes. The difference in the performance between the analyzed species could be related to factors that were not included in our models like transcriptional regulations or mRNA stability. Since it is documented that codon usage is linked with factors like mRNA stability, further studies adding this factor could be relevant (Hanson and Coller 2018).

Measures like CAI have the disadvantage that results can vary when different reference sets are employed (Bahiri-Elitzur and Tuller 2021). When the new index was evaluated by a reference dataset enriched with genes of lower expression we observed a decrease in the correlation, a behavior that could be related to dicodon contributions since when this was explored we observed that values of dicodon adaptiveness (*a*_d_) have an extensive distribution that can be associated with the poor performance. To overcome this, we implemented a square transformation over our weighted contributions that let us improve the correlations, even when all sequences were included in the reference set. We also observe that this strategy decreases the difference among sequences of different lengths.

Interestingly, our strategy was able to explain the contributions of dicodons that could not be explained by the single-codon approach. In this sense, we could mention 17 dicodons (Gamble *et al.* 2016) with confirmed translational inhibition, 15 of them with low values of *f*_d_. In accordance with those observations, we proposed other seven dicodons with low *f*_d_ being potential targets for experimental studies since this could be contributing to inhibiting protein translation, taking into account that translational inhibitory study of Gamble *et al.* (2016) does not include all possible hexamers. The improvement observed by introducing pairs of consecutive codons suggests that the order of the codons highlights the role of the translocation time of the ribosome between two consecutive codons during transcript translation (Diambra 2017). This translocation time could be a determinant of the ribosomal pauses program associated with proper protein folding (Komar 2009, Plotkin and Kudla 2011, McCarthy *et al.* 2017). In this context, our dicodon-based index is a promising tool applicable in biotechnology fields like codon pair deoptimization for virus attenuation and dicodon optimization for improving protein expression (Coleman *et al.* 2008).

Next, we evaluated the weighted dicodons index in a multicellular organism. Although some single-codon approaches with predictive power were documented (Sahoo *et al.* 2019), our weighted strategy improves codon indexes approaches when it was evaluated in *D.melanogaster*. However, the fact that CUB’s influence on gene expression is variable across tissues cannot be ignored. This particularity makes applying expression indexes in multi-cellular organisms, from its whole-body expression data, more difficult (Payne and Alvarez-Ponce 2019). Recently, tissues specifics metric for codon usage was developed (Allen *et al.* 2022). In this sense, our weighted dicodons approach could be expanded on the bases of tissues specifics data in future studies.

In conclusion, in this work, we improved the performance of the well-known CAI by using dicodon contributions. Additionally, we have implemented a weighted strategy for dicodon contributions taking into account the expression level that improved the correlations even further. Our dicodon index was evaluated on experimental unicellular models as *E.coli*, *T.gondii*, and *S.cerevisiae* outperforming codon-based indexes. Furthermore, our index was put to the test on a multi-cellular model organism like *D.melanogaster* showing better performance than CAI, although factors like tissue-specific CUB needed to be taken into account for applying expression indexes in this kind of model organisms.

## Supplementary Material

btad380_Supplementary_Data

## Data Availability

The coding sequences, transcript profiles and scripts used in this article are available in Zenodo, at https://dx.doi.org/10.5281/zenodo.7738276.
