# Supplementary material for: Dicodon-based measures for modeling gene expression
Source: Bioinformatics. 2023 Jun 12;39(6):btad380. doi: 10.1093/bioinformatics/btad380 (PMC10287933; doi:10.1093/bioinformatics/btad380)
Supplement: btad380_Supplementary_Data [file btad380_supplementary_data.zip › Suppmat.pdf]

Dicodon-based measures for modeling gene expression

Andres M. Alonso[1,3],
and Luis Diambra[2,3,*]

[1]INTech, Universidad Nacional de San Martin, Chascomus, Argentina
[2]CREG, Universidad Nacional de La Plata, La Plata, Argentina
[3]CONICET
[*]corresponding author: ldiambra@gmail.com

Supplementary Material

Supplementary Table Legends

Table S1: List of the different samples/conditions for the four organisms used in this paper. The last column provides direct links for the expression profiles used.
Table S2: Pearson correlation coefficients obtained for the four schemes (weighted codons, no-weighted codons, weighted dicodons, and no-weighted dicodons) using transcript sequences with three different percentiles (80, 90, and 97). Columns (C, F, and I) depict the average correlations obtained for short and long sequences, columns (D, G, and J) are differences between these means. Columns (E, H, and K) list the p-values obtained with the paired T-test. The table below is similar but obtained using squared contributions. Green cells are indicating the cases where differences among correlations from short and long sequences are not significant.

Supplementary Figures

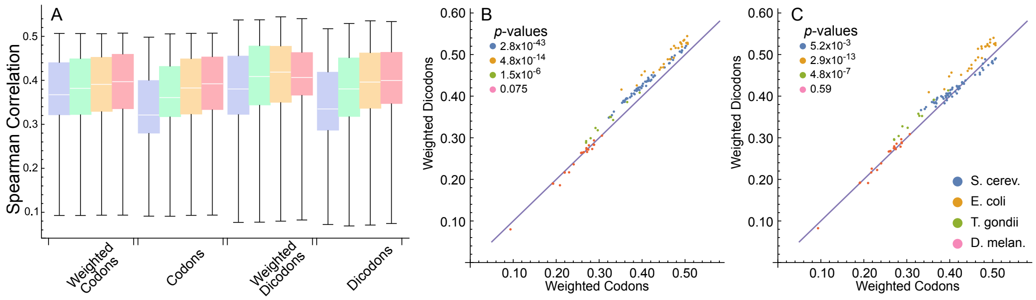

Fig. Supp. 1: Spearman's rank correlation coefficients between the expression indexes and expression level in yeast. They correspond to four schemes (weighted codons, no-weighted codons, weighted dicodons, and no-weighted dicodons) using transcript sequences with four different percentiles. The Spearman's rank correlation coefficients are computed for each sample independently (A). For comparison among different schemes, we plot the Spearman correlations obtained for each sample for weighted codons vs. weighted dicodons (percentile 95) (B), and weighted codons vs. weighted dicodons (percentile 97) (C). Solid-line is the identity line and the p-values were obtained with the paired T-test.

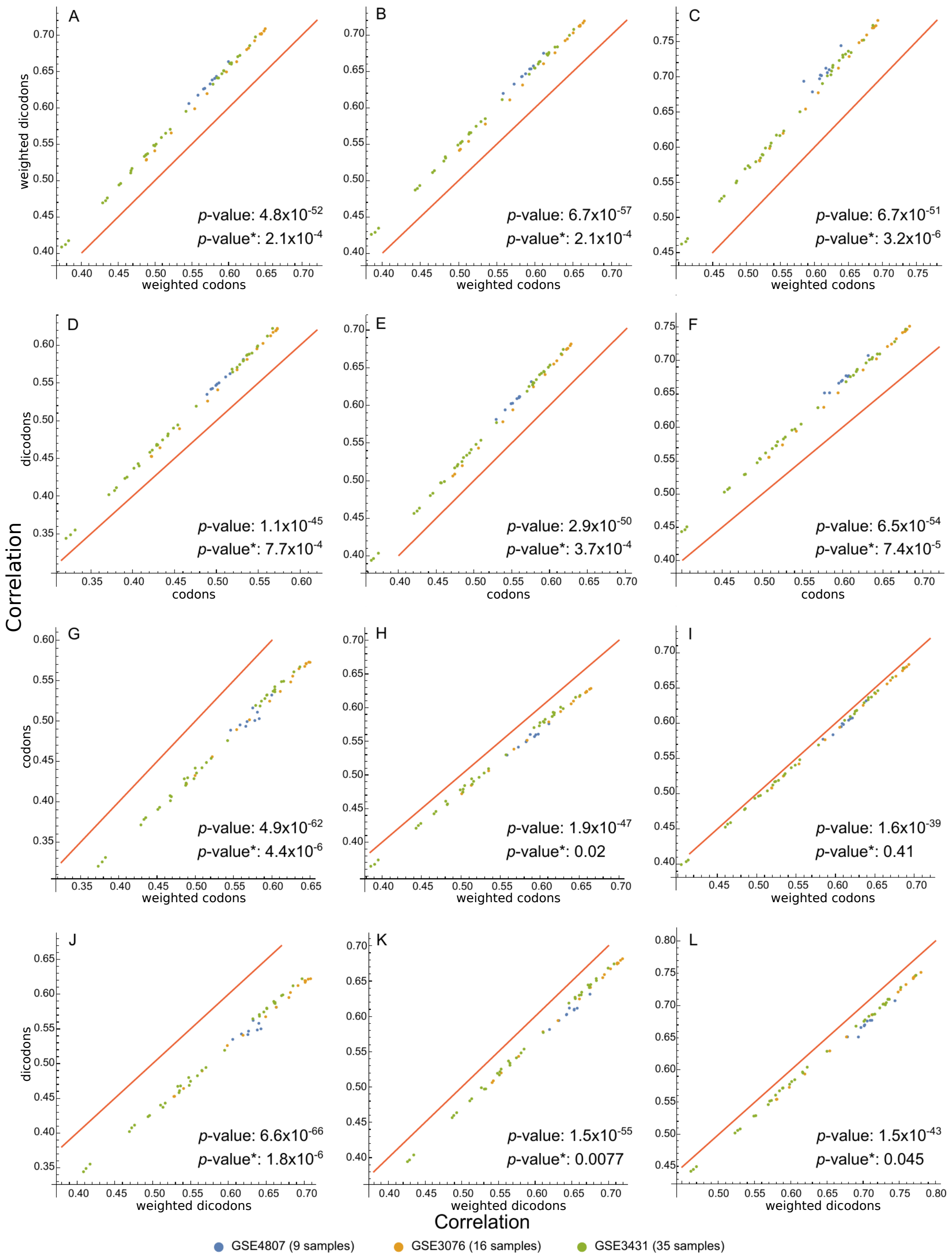

Fig. Supp. 2: Raster plots of the Pearson correlation coefficients from each sample of *S. cerevisiae* using different indexes. The different reference gen sets used are organized vertically: panels A, D, G, and J correspond to reference gene set S_{80}, panels B, E, H and K correspond to reference gene set S_{90}, and C, F, I, and L correspond to reference gene set S_{97}. Panels A-C depict the comparisons among correlations obtained with weighted codons (horizontal axes) vs. weighted dicodons (vertical axes). Panels D-F depicts the comparison among codons (horizontal axes) vs. dicodons (vertical axes). Panels G-I depicts the comparison among weighted codons (horizontal axes) vs. codons (vertical axes). Panels J-L depicts the comparison among weighted dicodons (horizontal axes) vs. dicodons (vertical axes). Each dot represents a different sample, while solid lines are the identity line. The p-values were obtained with the paired T-test, while the p-values* were obtained with the Mann-Whitney test.

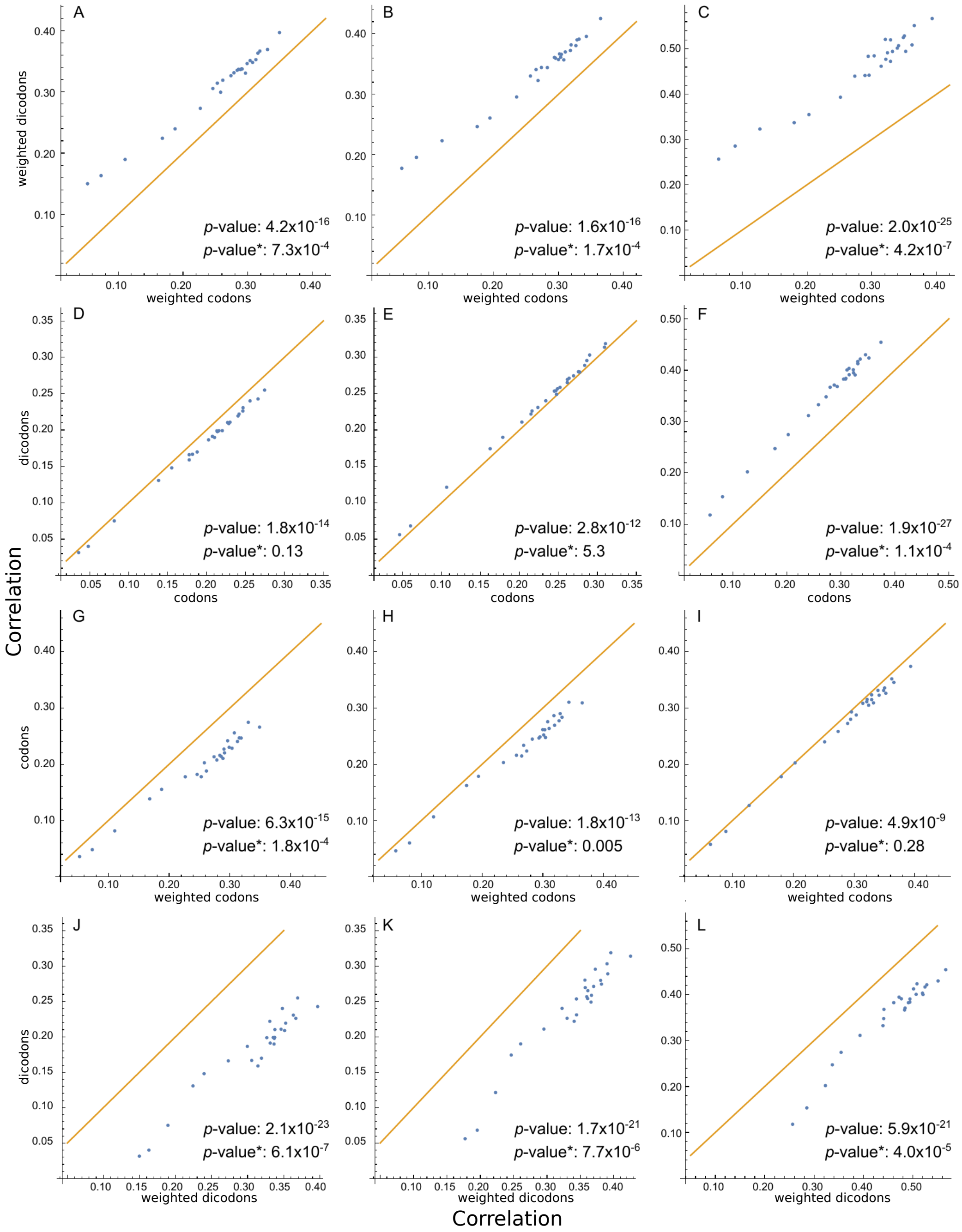

Fig. Supp. 3: Raster plots of the Pearson correlation coefficients from each sample of *E. coli* using different indexes. The different reference gen sets used are organized vertically: panels A, D, G, and J correspond to reference gene set S_{80}, panels B, E, H and K correspond to reference gene set S_{90}, and C, F, I, and L correspond to reference gene set S_{97}. Panels A-C depict the comparisons among correlations obtained with weighted codons (horizontal axes) vs. weighted dicodons (vertical axes). Panels D-F depicts the comparison among codons (horizontal axes) vs. dicodons (vertical axes). Panels G-I depicts the comparison among weighted codons (horizontal axes) vs. codons (vertical axes). Panels J-L depicts the comparison among weighted dicodons (horizontal axes) vs. dicodons (vertical axes). Each dot represents a different sample, while solid lines are the identity line. The p-values were obtained with the paired T-test, while the p-values* were obtained with the Mann-Whitney test.

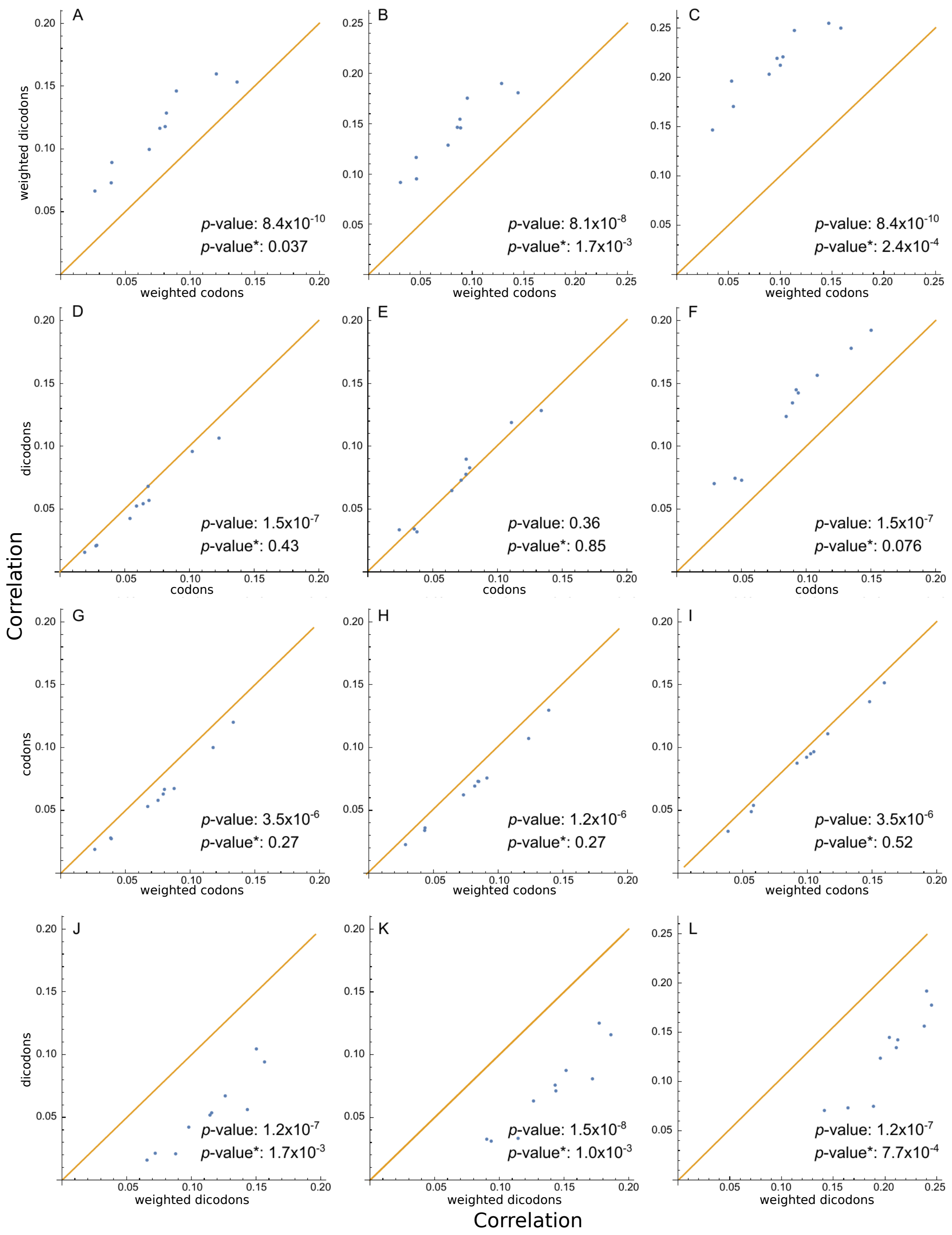

Fig. Supp. 4: Raster plots of the Pearson correlation coefficients from each sample of *T. gondii* using different indexes. The different reference gen sets used are organized vertically: panels A, D, G, and J correspond to reference gene set S_{80}, panels B, E, H and K correspond to reference gene set S_{90}, and C, F, I, and L correspond to reference gene set S_{97}. Panels A-C depict the comparisons among correlations obtained with weighted codons (horizontal axes) vs. weighted dicodons (vertical axes). Panels D-F depicts the comparison among codons (horizontal axes) vs. dicodons (vertical axes). Panels G-I depicts the comparison among weighted codons (horizontal axes) vs. codons (vertical axes). Panels J-L depicts the comparison among weighted dicodons (horizontal axes) vs. dicodons (vertical axes). Each dot represents a different sample, while solid lines are the identity line. The p-values were obtained with the paired T-test, while the p-values* were obtained with the Mann-Whitney test.

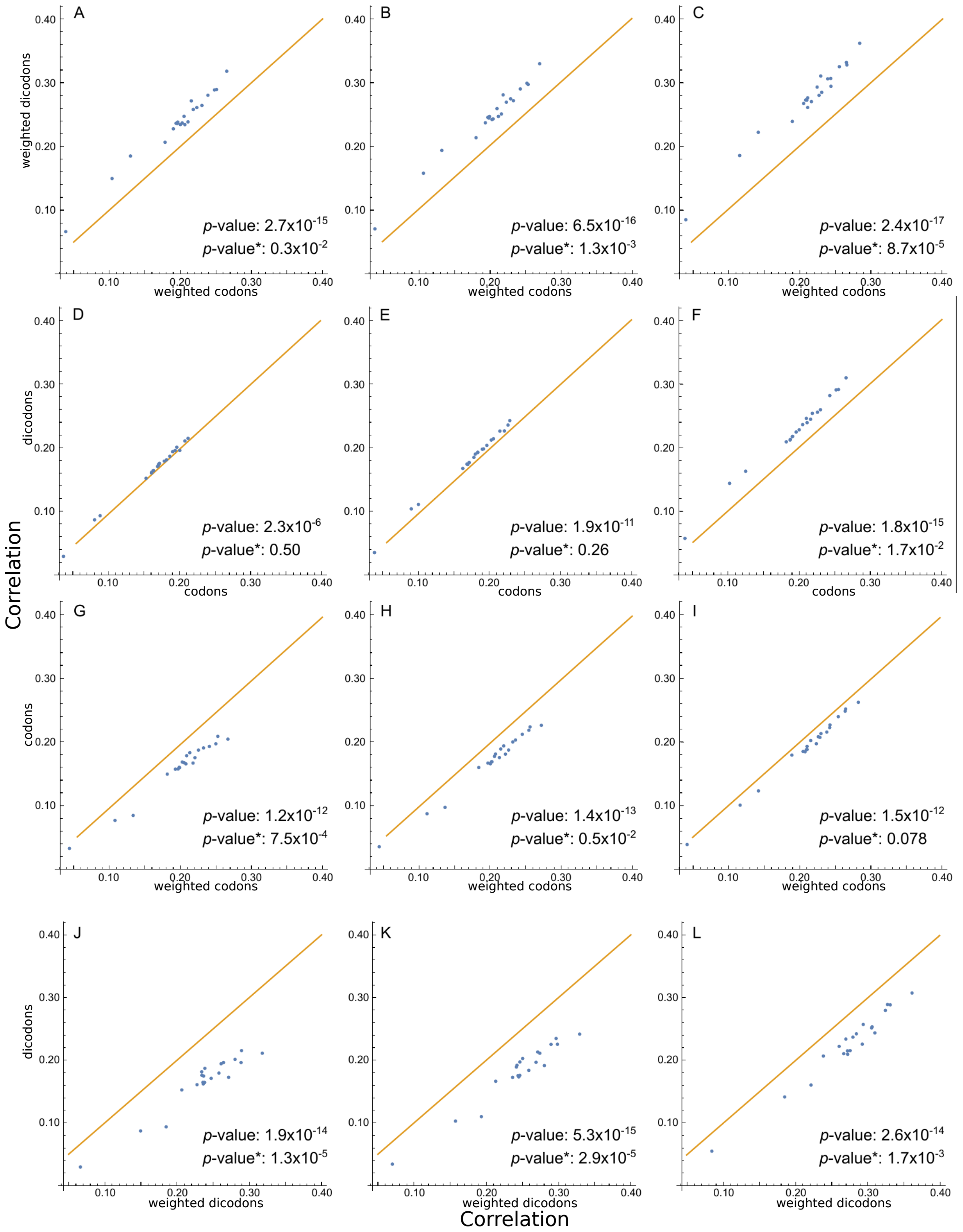

Fig. Supp. 5: Raster plots of the Pearson correlation coefficients from each sample of *D. melanogaster* using different indexes. The different reference gen sets used are organized vertically: panels A, D, G, and J correspond to reference gene set S_{80}, panels B, E, H and K correspond to reference gene set S_{90}, and C, F, I, and L correspond to reference gene set S_{97}. Panels A-C depict the comparisons among correlations obtained with weighted codons (horizontal axes) vs. weighted dicodons (vertical axes). Panels D-F depicts the comparison among codons (horizontal axes) vs. dicodons (vertical axes). Panels G-I depicts the comparison among weighted codons (horizontal axes) vs. codons (vertical axes). Panels J-L depicts the comparison among weighted dicodons (horizontal axes) vs. dicodons (vertical axes). Each dot represents a different sample, while solid lines are the identity line. The p-values were obtained with the paired T-test, while the p-values* were obtained with the Mann-Whitney test.

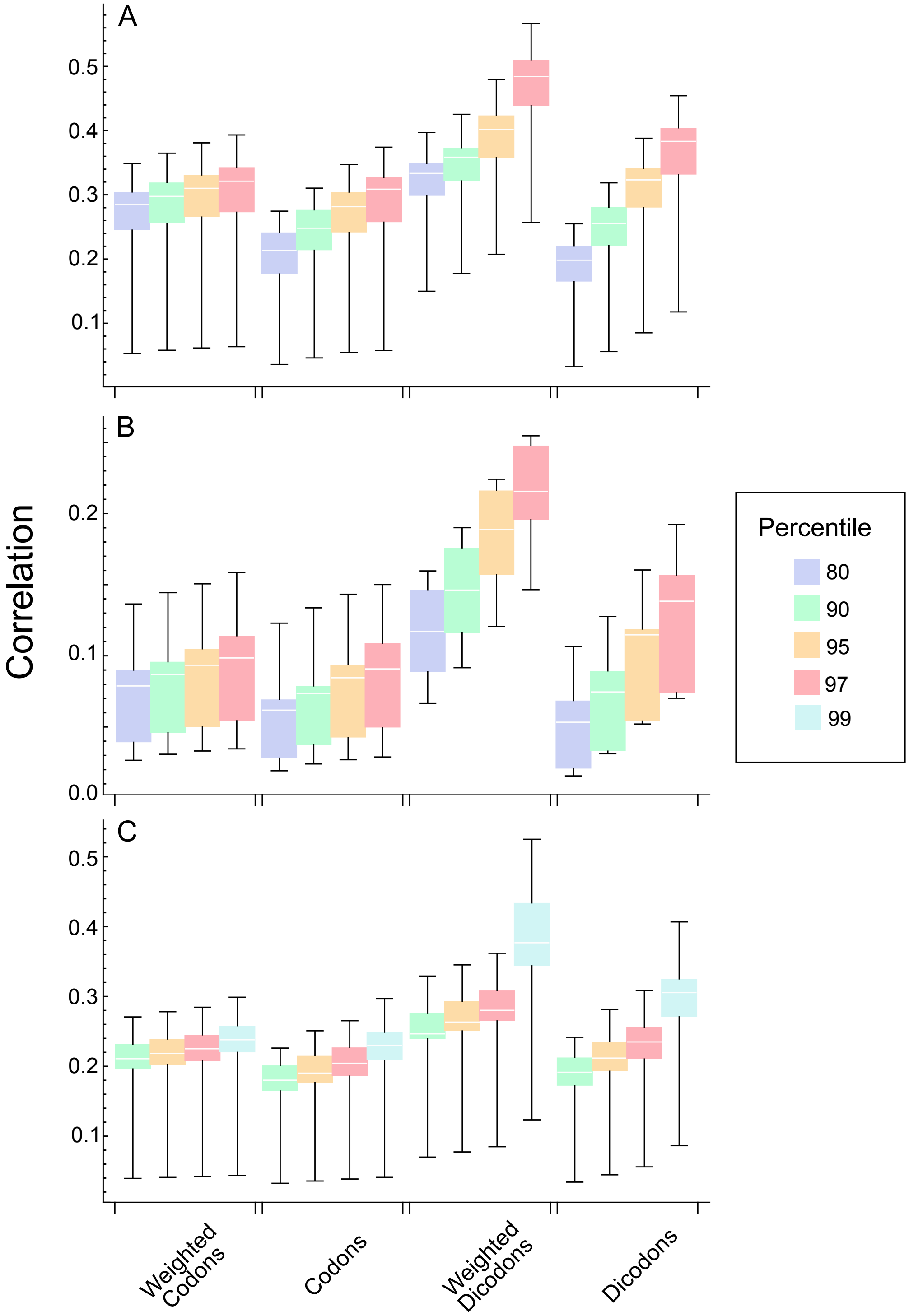

Fig. Supp. 6: Pearson's correlation coefficients between the expression indexes and expression levels obtained from *E. coli*(A), *T. gondii* (B), and *D. melanogaster* (C). They correspond to four schemes (weighted codons, no-weighted codons, weighted dicodons, and no-weighted dicodons) using transcript sequences with different percentiles, as indicated by color boxes in the panel legend. The correlation coefficients are computed for each sample independently and for each organism independently.
